# DendroX: multi-level multi-cluster selection in dendrograms

**DOI:** 10.1186/s12864-024-10048-0

**Published:** 2024-02-02

**Authors:** Feiling Feng, Qiaonan Duan, Xiaoqing Jiang, Xiaoming Kao, Dadong Zhang

**Affiliations:** 1 Department of Biliary Tract Surgery I, Eastern Hepatobiliary Surgery Hospital, Shanghai, China; 2 Department of Clinical and Translational Medicine, 3D Medicines Inc., Shanghai, China; 3 Research Institute of General Surgery, Jinling Hospital, Affiliated Hospital of Medical School, Nanjing University, Nanjing, China

**Keywords:** Dendrogram, Cluster analysis, LINCS L1000, Natural medicine

## Abstract

**Background:**

Cluster heatmaps are widely used in biology and other fields to uncover clustering patterns in data matrices. Most cluster heatmap packages provide utility functions to divide the dendrograms at a certain level to obtain clusters, but it is often difficult to locate the appropriate cut in the dendrogram to obtain the clusters seen in the heatmap or computed by a statistical method. Multiple cuts are required if the clusters locate at different levels in the dendrogram.

**Results:**

We developed DendroX, a web app that provides interactive visualization of a dendrogram where users can divide the dendrogram at any level and in any number of clusters and pass the labels of the identified clusters for functional analysis. Helper functions are provided to extract linkage matrices from cluster heatmap objects in R or Python to serve as input to the app. A graphic user interface was also developed to help prepare input files for DendroX from data matrices stored in delimited text files. The app is scalable and has been tested on dendrograms with tens of thousands of leaf nodes. As a case study, we clustered the gene expression signatures of 297 bioactive chemical compounds in the LINCS L1000 dataset and visualized them in DendroX. Seventeen biologically meaningful clusters were identified based on the structure of the dendrogram and the expression patterns in the heatmap. We found that one of the clusters consisting of mostly naturally occurring compounds is not previously reported and has its members sharing broad anticancer, anti-inflammatory and antioxidant activities.

**Conclusions:**

DendroX solves the problem of matching visually and computationally determined clusters in a cluster heatmap and helps users navigate among different parts of a dendrogram. The identification of a cluster of naturally occurring compounds with shared bioactivities implicates a convergence of biological effects through divergent mechanisms.

**Supplementary Information:**

The online version contains supplementary material available at 10.1186/s12864-024-10048-0.

## Background

A cluster heatmap consists of a heatmap and two dendrograms in its basic form [1]. While it does not specify the number of clusters on each dimension, users can divide the dendrograms at a certain level to obtain clusters. The cut-off levels can be selected manually or by a statistical method [2–4]. If selected manually, the process usually involves matching a well-defined color patch in the heatmap to a well aligned sub-tree in the dendrogram and then determining the level at which the sub-tree resides. Multiple cut-offs are needed if the clusters are located at different levels. Several software packages have been developed to generate static cluster heatmaps [5–7], including the pheatmap in R and the Seaborn in Python [8, 9]. Both provide utility functions to cut dendrograms at a certain level to obtain clusters, but fall short of helping identify the most appropriate cut, which makes manual assignment difficult especially for large heatmaps with complex dendrograms.

To assign clusters statistically, pvclust is the most popular method [2]. It assigns *p*-values to clusters in dendrograms through bootstrap resampling. It does not, however, visualizes the dendrograms along with a heatmap and highlight the significant clusters in distinct colors. This is where an interactive dendrogram can help. Moreover, it takes a very long time to run the statistical analysis on a large dendrogram, which can be impractical for an exploratory analysis with many trials.

Web-based tools have been developed to generate interactive cluster heatmaps [10–13]. However, they are mostly a duplication of the functions provided by existing packages and not scalable to large datasets because of the limited processing power of web browsers. Almost none offer the cluster-selection feature that helps match visually and computationally determined clusters. The only exception is InChlib [14], which allows selection of one cluster at a time in the row dendrogram. InChlib, however, does not provide clustering on the column dimension, does not allow creation of distinctly colored child clusters, and lacks the ability to extract text labels from selected clusters for functional analysis. To improve on existing tools, we developed DendroX, a web app that enables multiple-cluster selection at different levels in a dendrogram and extraction of text labels from selected leaf nodes. Designed as a downstream tool to the pheatmap and Seaborn packages, it combines the processing power of offline packages with the interactive features of an online app.

The Library of Integrated Network-based Cellular Signatures (LINCS) program aims to profile molecular signatures of cell lines that are perturbed by chemical or genetic agents. The LINCS L1000 project specifically measures the gene expression changes of such cell lines using the L1000 technology and is a scaled-up version of the Connectivity Map project [15, 16]. The L1000 technology uses a reduced set of 978 genes to represent the whole transcriptome and employs a machine learning model to infer the expression of the rest transcriptome. It has produced over one million gene expression profiles of cell lines treated with tens of thousands of different chemical or genetic agents. Some of these agents are naturally occurring compounds that have no well-defined targets and can potentially affect multiple pathways in a cell line, which makes it difficult to characterize their biomedical activities. The LINCS L1000 dataset offers a unique window to explore their aggregated biological effects on the transcriptomic level.

## Method

### Implementation

DendroX was developed as a front-end only app in which the submitted data are processed within the browser and not sent to a remote server. The React framework [17], a JavaScript library for building modularized user interfaces, was used to structure the app into two views: an input view to submit data and a visualization view to draw the dendrogram. The visualization was created using the D3 library [18], and the image editing function was borrowed and customized from the react-image-crop library. The D3 library is used to manipulate dynamic web graphics and the react-image-crop library provides a React module for editing static images. The react-icons package, which is a collection of customized HTML icons, was used to generate the iconized buttons in the app. Helper functions were provided in Python and R to extract linkage matrices from cluster heatmap objects and convert them into JSON files that serve as input to the app. The session saving function were implemented in two ways. One is to download the internal representation of the session as a JSON file and the other is to save it in the browser’s local storage using IndexedDB [19]. The DendroX Cluster program was developed using the Python Eel library, which takes advantage of the UI infrastructure of JavaScript and the data analysis capability of Python to build standalone computer programs. A table of packages and libraries used in developing the DendroX app was provided in the supplementary (Table S1).

### Case study

The LINCS L1000 gene expression data were downloaded from Gene Expression Omnibus. Differential expression signatures were calculated for each experiment using the characteristic direction method [20]. Average cosine distance (ACD) between the replicates of an experiment was used to represent its strength [21] The smaller the ACD, the larger the bioactivity of a chemical compound is. The distribution of ACDs is shown in supplementary Figure S1. A compound might be tested in multiple experiments and its signature was calculated as the average of these experiments’ signatures. We only selected named compounds that were tested in at least 10 experiments with an average ACD of less than 0.9. The 978-gene signature matrix of these compounds was z-score standardized along the column dimension and clustered using the average linkage function. The row distance metric was set to cosine and the column metric to correlation distance. The cluster heatmap image was created using the Python Seaborn library (Supplementary Figure S2, showing the row and column dendrograms of the cluster heatmap) with its object converted into a JSON file using the get_json function provided by DendroX. The JSON file and the cluster heatmap image were submitted in DendroX to create an interactive cluster heatmap.

## Results

### Prepare input files

The required input to the app is a JSON file that stores the linkage matrix of a dendrogram. JSON denotes JavaScript Object Notation, a text file format consisting of arrays and key-value pairs. It is a flexible yet standard format to save data and easily processed in a web browser. An input JSON file can be created in two ways. First, it can be created programmatically using the R or Python function provided by us. These functions take the return value of the Python seaborn.clustermap or the R pheatmap function as argument and outputs a JSON file. The links to the functions and their examples can be found in the help section of the input view. We have wrapped the functions into R and Python packages that can be easily installed in the users’ computing environment. The DendroX app also accepts a JPEG or PNG file as an optional input. It should be an image of the heatmap associated with the input dendrogram, usually the cluster heatmap figure generated by the seaborn.clustermap or pheatmap function.

**Fig. 1:**
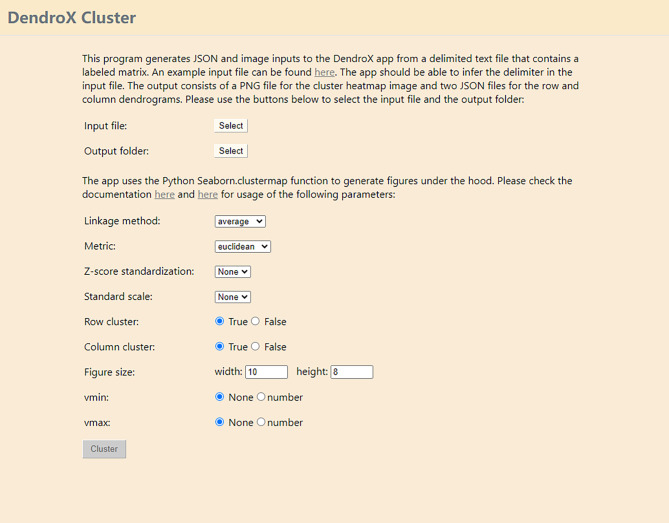
A screenshot of the user interface of the DendroX Cluster program

Second, the JSON file can be created in a few clicks using the DendroX Cluster program (Fig. 1). The program is a standalone graphic user interface that run locally on users’ computers. It takes a data matrix as input, runs the cluster heatmap function and outputs input files for the DendroX app. The input matrix should have row and column labels and be stored in a delimited text file. The delimiter of the file is inferred automatically by the program. After the input file is selected, the output folder is automatically set to the folder of the input file. Users can choose another folder by clicking on the folder selection button. The program provides several options to customize the cluster heatmap. The parameters of these options are the same as provided by the Python seaborn.clustermap function. After configuring the parameters, click on the cluster button to run the cluster function. By default, the program generates three files in the output folder with the name of the input file as prefix. Two are JSON files for the row and column dendrograms and one a PNG file of the cluster heatmap image. The image will also be displayed below the cluster button for quick review.

### Input view

**Fig. 2:**
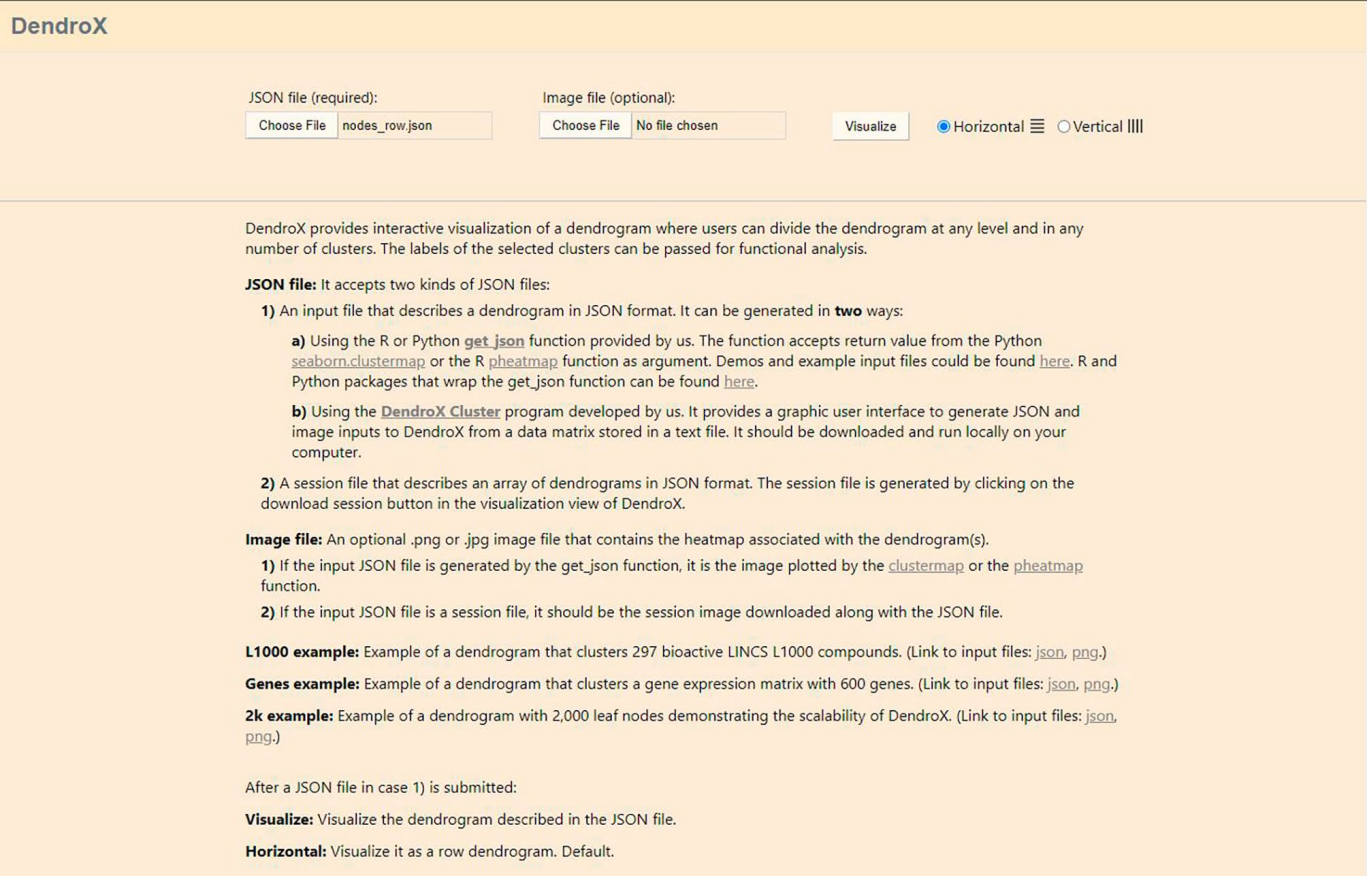
A screenshot of the input view. It shows the input view with an example JSON file and image file loaded. The example data is adapted from [22]

After a JSON file is submitted, a “Visualize” button and two radio buttons will appear (Fig. 2). The radio buttons allow switching between the horizontal and vertical layouts. The dendrogram will be a row dendrogram if “Horizontal” is selected and a column one if “Vertical” is selected. Click on the “Visualize” button to navigate to the visualization view. If a JPEG or PNG image file is also submitted, it will be visualized besides the dendrogram in the visualization view.

### Visualization view

**Fig. 3:**
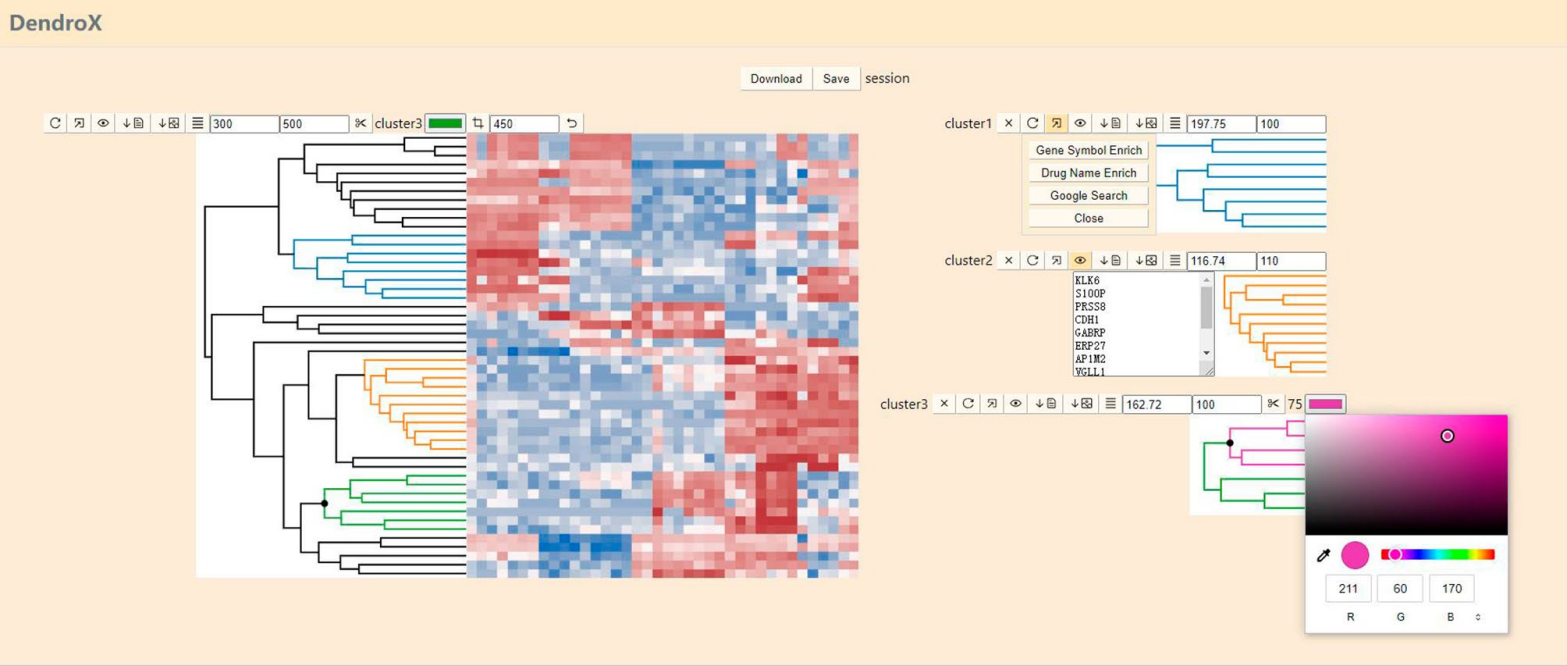
A screenshot of the visualization view. It visualizes the data submitted in Fig. 2

In the visualization view (Fig. 3), the data in the JSON file are visualized as an interactive dendrogram. To highlight a cluster, an operator moves the cursor over a non-leaf node in the dendrogram. Information about the cluster will be shown including the id, the name (if it exists) and the number of leaf nodes of that cluster. To select the cluster, the operator clicks on the non-leaf node. To unselect it, the operator clicks again. To unfocus a selected cluster, the operator clicks on any white space. The app will automatically assign a color to a selected cluster. To change the assigned color, the operator clicks on the color box. To create a child dendrogram from the cluster, the operator clicks on the scissor button. The text between the scissor and the color box is the id or name of the cluster.

The operator clicks on the view button to open a text box that lists the labels of the leaf nodes. These labels can be interpreted through external tools. Click on the external tool button to open a panel of external tools. If the labels are gene symbols, press the Gene Symbol Enrich button to pass the labels to the Enrichr app [23] for gene-set enrichment analysis. If the labels are drug/compound names, press the Drug Name Enrich button to pass the labels to the DrugEnrichr app [24] for drug name-set enrichment analysis. The DrugEnrichr app is a variant of the Enrichr app that uses drug name sets instead of gene sets for enrichment analysis run by the same algorithm. If users want to search the labels one by one in Google, press the Google Search button to open a tab for each searched label. Users have to choose “always allow pop-ups from this site” to use this feature.

If provided, the heatmap image will be placed on the right-hand side of the dendrogram in a horizontal layout and underneath it in a vertical layout. A crop button is implemented to align the heatmap with the dendrogram on the matched dimension. Three input boxes are implemented to adjust the widths and heights of the dendrogram and heatmap. Their sizes will always be the same on the matched dimension. As the heatmap is a static image, the app is scalable and has been tested on dendrograms with more than 10,000 leaf nodes.

### Session saving

There are two ways to save a session. The first is to download the session as a JSON file that can be shared with others to reconstruct the session in DendroX. If a heatmap image is provided, the cropped image will be saved as a PNG file along with the JSON file. To load the saved session, simply submit the JSON and the PNG files in the input view as standard input files and click on the Load session button that will appear after they were submitted. The second way to save a session is to click on the save session button on top of the cluster heatmap, which will save the session in the browser’s local storage. A session link will be created in the input view that contains the name of the session and the time it was last saved. Click on the session link to load the session. Click on the “remove” button next to the link to delete the session. On the right of the session saving buttons is an editable text area displaying the session name. Click on this area to change the session name.

### Case study

As a demonstration of the key functionalities of DendroX, we clustered and visualized the gene expression signatures of 297 bioactive chemical compounds in the LINCS L1000 dataset [15]. A gene expression signature is a vector of 978 components that represent how much each gene is perturbed from the control by a chemical agent in a given experimental setting. The gene expression signatures were calculated using the characteristic direction method [20, 25] that performs better than the commonly used z-score method [21, 26] in benchmarking. The compounds are selected by the criteria that each must have a valid drug name, a significance score passing a predefined threshold and at least 10 expression signatures. The final signature is the average of the expression signatures of a compound.

**Fig. 4:**
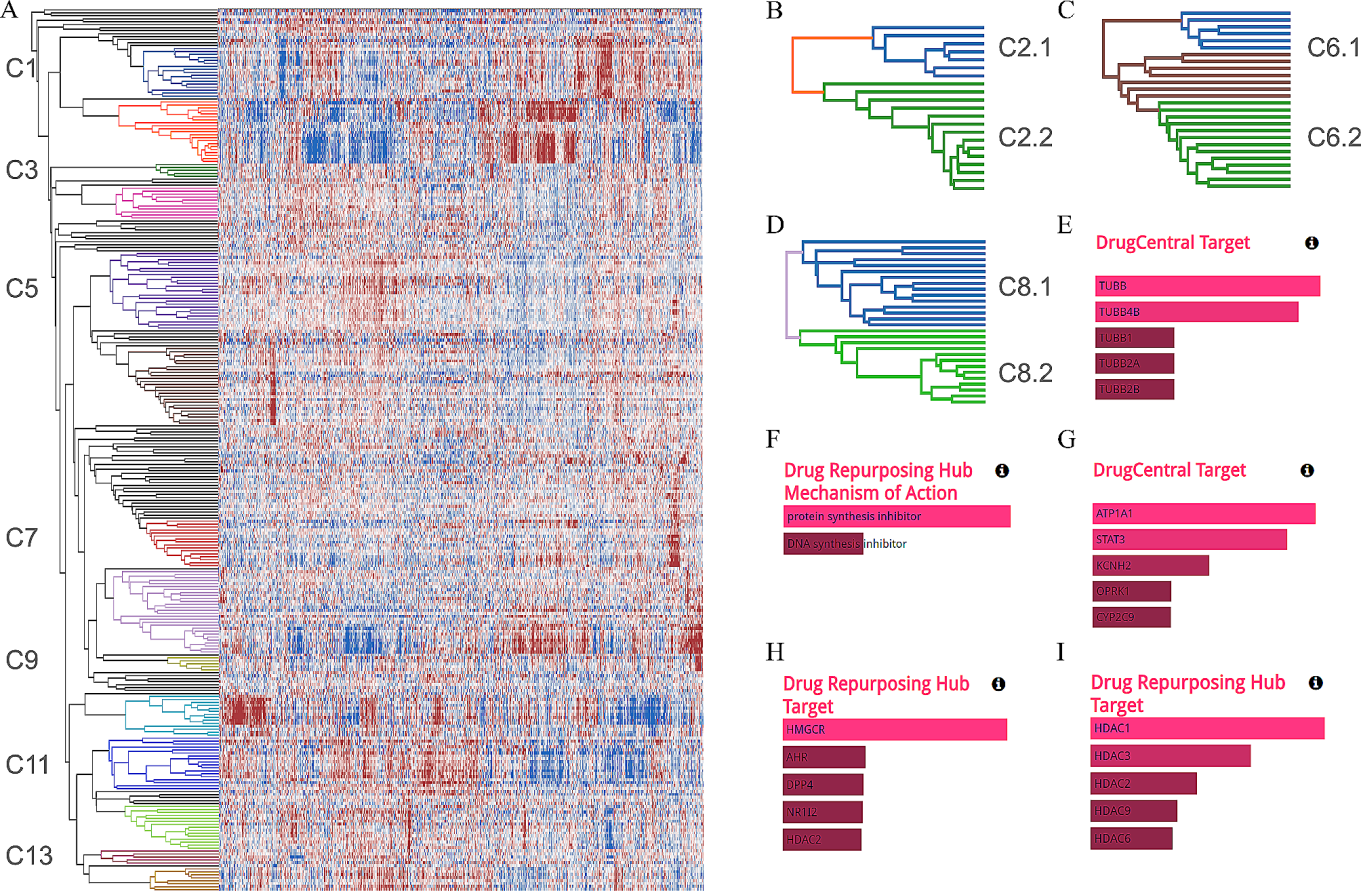
**A:** cluster heatmap of the 297 LINCS L1000 compound signatures. The 14 major clusters are colored and labeled. **B-D:** sub-clusters of cluster 2, 6 and 8. **E-I:** drug name-set enrichment analysis results of cluster 1, cluster 2.1, cluster 2.2, cluster 6.1 and cluster 10

Figure 4A shows the cluster heatmap of the 297 bioactive compounds visualized in DendroX. We identified 14 major clusters based on the structure of the dendrogram and the expression patterns in the heatmap (Table 1). A child dendrogram was created for each cluster and drug name-set enrichment analysis was performed on the labels of their leaf nodes. Cluster 1, 3, 5, 9, 10, 11, 12, 13 and 14 are clearly enriched in a distinct single class of targets that reveal their common MOAs (Fig. 4E and I as examples). In contrast, both cluster 11 and cluster 12 are enriched in topoisomerase inhibitors. Google search on their compound names shows that chemicals in cluster 11 are also enriched in kinase inhibitors like dinaciclib, dorsomorphin, staurosporine, lestaurtinib and rebastinib while the majority of compounds in cluster 12 are chemotherapy drugs classified as antimetabolites. So we labeled cluster 11 as TOP/kinase and cluster 12 as antimetabolite.

Cluster 2 (Fig. 4B) is enriched in a mixture of targets and comprises two branches. A child dendrogram was created for each branch and drug name-set enrichment analysis was performed on each sub-cluster. The results show that cluster 2.1 is enriched in protein synthesis inhibitors and cluster 2.2 in ATPase inhibitors (Fig. 4F and G). Similarly, we created child dendrograms for cluster 6 and 8 (Fig. 4C and D) that are enriched in a mix of targets according to the structure of their dendrograms. While drug name-set enrichment analysis suggested cluster 6.1 is enriched in HMGCR inhibitor (Fig. 4H) and cluster 8.2 in proteasome inhibitor, it did not provide clear indications on what targets the other child clusters are enriched in. We examined the drug names of these child clusters in Google and found that cluster 6.2 is enriched in psychiatric drugs and cluster 8.1 in naturally occurring compounds.

**Table 1:** The 17 clusters and the number of compounds in them

Cluster	Label	Count
C1	TUBB	17
C2.1	Protein synthesis	7
C2.2	ATPase	14
C3	VD	5
C4	Corticoid	11
C5	MAPK	26
C6.1	HMGCR	6
C6.2	Psychiatric	13
C7	Antibiotics	16
C8.1	Naturally occurring	15
C8.2	Proteasome	13
C9	HSP	5
C10	HDAC	14
C11	TOP/Kinase	18
C12	Antimetabolite	15
C13	Aurora	5
C14	PI3K/mTOR	8

Cluster 8.1 consists of a diverse set of naturally occurring compounds with different known MOAs. It is interesting to see their expression patterns converged and clustered together in the dendrogram. We searched these compounds in literature and found that despite their diversity they share the same anticancer and anti-inflammatory biological effects and are related to oxidative stress (Table 2). Their similar gene expression patterns may be a reflection of these shared effects. Among them, guggulsterone, brazilin, parthenolide, butein, isoliquiritigenin and celastrol are active gradients of herb-based therapies that have been used in traditional medicine for hundreds of years [27–32]. Some in the cluster like brazilin and butein are classified as antioxidants while others like parthenolide and piperlongumine are classified as oxidative stress inducers. This contradiction may be explained by the findings in fenretinide that the compound exerts antioxidant effects at low concentration and promotes ROS accumulation at high concentration in a context dependent manner [33]. Two compounds in this list are synthetic chemicals with BNTX being a standard delta opioid receptor antagonist and Elesclomol a copper ionophore and first-in-class HSP70 inducer [34]. BNTX was found to suppress immune functions and sensitize cancer cells to apoptosis [35, 36] and Elesclomol to induce oxidative stress and cuproptosis [37]. Our results suggest both drugs have potentially multiple targets and could be further explored for their anti-inflammatory abilities.

**Table 2:** The 15 compounds in cluster 8.1 and their known biological effects in literature

Compound Name	Biological Effects	Reference
Guggulsterone	Anticancer, anti-inflammatory	[38]
Capsazepine	Anticancer, anti-inflammatory, ROS pathway	[39]
Piceatannol	Anticancer, anti-inflammatory, antioxidant	[40]
Fenretinide	Anticancer, anti-inflammatory, antioxidant or oxidative stress inducer	[33, 41, 42]
Xanthohumol	Anticancer, anti-inflammatory	[43]
Elesclomol	Anticancer, oxidative stress inducer	[37]
Brazilin	Anticancer, anti-inflammatory, antioxidant	[44, 45]
Cerulenin	Anti-inflammatory	[46]
Parthenolide	Anticancer, anti-inflammatory, oxidative stress inducer	[47, 48]
Butein	Anticancer, anti-inflammatory, antioxidant	[30]
Isoliquiritigenin	Anticancer, anti-inflammatory, antioxidant	[49–51]
Piperlongumine	Anticancer, anti-inflammatory, oxidative stress inducer	[52]
Thiostrepton	Anticancer, oxidative stress inducer	[53, 54]
BNTX (7-benzylidene naltrexone)	Immunosuppressive, apoptosis sensitizer	[35, 36]
Celastrol	Anticancer, anti-inflammatory, antioxidant	[32]

Cluster 4 and 7 were not enriched into any target class and dividing them into child dendrograms did not help. We then searched their compound names in Google and found cluster 4 is enriched in corticoids and cluster 7 in antibiotics. In total, we identified 17 clusters with distinct expression patterns and listed them in Table 1. The detailed table that includes the compound names in each cluster can be found in supplementary Table S2. Users can review this case study by clicking the L1000 example button in the input view.

Besides the LINCS L1000 case study, we also conducted another case study on gene expression data in the supplementary. It demonstrates how to visualize hierarchically clustered gene expression matrices in DendroX and perform gene-set enrichment analysis on the clustered genes.

## Discussion

Previous work has visualized LINCS L1000 chemical signatures in a t-SNE plot [15], in a UMAP projection [55] or in a firework display [26]. Such visualization methods represent each signature as a dot and use the distance between the dots to represent their similarity. A cluster heatmap on the other hand enables the direct comparison of the gene expression patterns on the single gene level. It can reveal more mechanistically complex clusters like cluster 8.1 that are not previously reported based on the structure of the dendrogram and the expression patterns in the heatmap. The compounds in cluster 8.1 should also be in proximal positions in a t-SNE or UMAP plot. But when researchers checked their MOAs, no common biology was found and they probably refrained from defining them as a valid cluster. In contrast, in an interactive cluster heatmap like DendroX, we can clearly see that the expression pattern of cluster 8.1 is different from other clusters and have more confidence in defining it as a biologically meaningful cluster.

Cluster 8.1 is closely located to cluster 8.2 which is a cluster of proteasome inhibitors. Literature search suggests that some of the naturally occurring compounds in cluster 8.1 are proteasome inhibitors including celastrol [56], thiostrepton [57] and xanthohumol [58]. The cluster also includes parthenolide that inhibits ubiquitin-specific peptidase 7 (USP7) [59] and capsaicin that induces proteasome system dysfunction [60]. These evidence suggest that the shared bioactivities of the compounds in cluster 8.1 may be related to their actions on the proteasome pathway. However, the gene expression pattern of cluster 8.1 is also quite distinct from that of cluster 8.2. Other pathways should be involved in producing this unique pattern due to the multi-target nature of the natural compounds.

## Conclusions

The DendroX app is developed to solve the problem of matching visually and computationally determined clusters in cluster heatmaps. It implements multi-level multi-cluster selection to enable direct comparison of multiple clusters in one place. As a case study, we identified a cluster of naturally occurring compounds with similar gene expression patterns and shared biomedical activities in DendroX. It demonstrates the utility of our app in aiding biological discoveries.

### Availability and requirements

Project name: DendroX.

Project home page: https://github.com/frlender/DendroX.

Operating system(s): Platform independent.

Programming language: TypeScript.

Other requirements: None.

License: MIT.

Any restrictions to use by non-academics: None.

### Electronic supplementary material

Below is the link to the electronic supplementary material.

Supplementary Material 1

Supplementary Material 2

Supplementary Material 3

## Data Availability

The app is available at: https://frlender.github.io/dendrox-app/. The data sets and code that replicate the results in this article are available on Github at the following URLs: https://github.com/frlender/DendroX/tree/main/public; https://github.com/frlender/DendroX/tree/main/input_demo; https://github.com/frlender/DendroX/tree/main/asset. R and Python packages that wrap the get_json function to generate input files: https://github.com/frlender/dendrox-util. The source code and executables of the DendroX Cluster program can be found and downloaded here: https://github.com/frlender/dendrox-cluster.

## Abbreviations

LINCS: The library of integrated network-based cellular signatures program
JSON: JavaScript object notation
ACD: Average cosine distance
JPEG: Joint photographic experts group
PNG: Portable network graphics
HTML: Hypertext markup language
MOA: Mechanism of action
t-SNE: t-distributed stochastic neighbor embedding
UMAP: Uniform manifold approximation and projection
ROS: Reactive oxygen species
BNTX: 7-benzylidene naltrexone
