# Supplementary material for: DendroX: multi-level multi-cluster selection in dendrograms
Source: BMC Genomics. 2024 Feb 2;25:134. doi: 10.1186/s12864-024-10048-0 (PMC10835886; doi:10.1186/s12864-024-10048-0)
Supplement: Supplementary file 3 — Supplementary Material 3 [file 12864_2024_10048_MOESM3_ESM.docx]

**Figures**


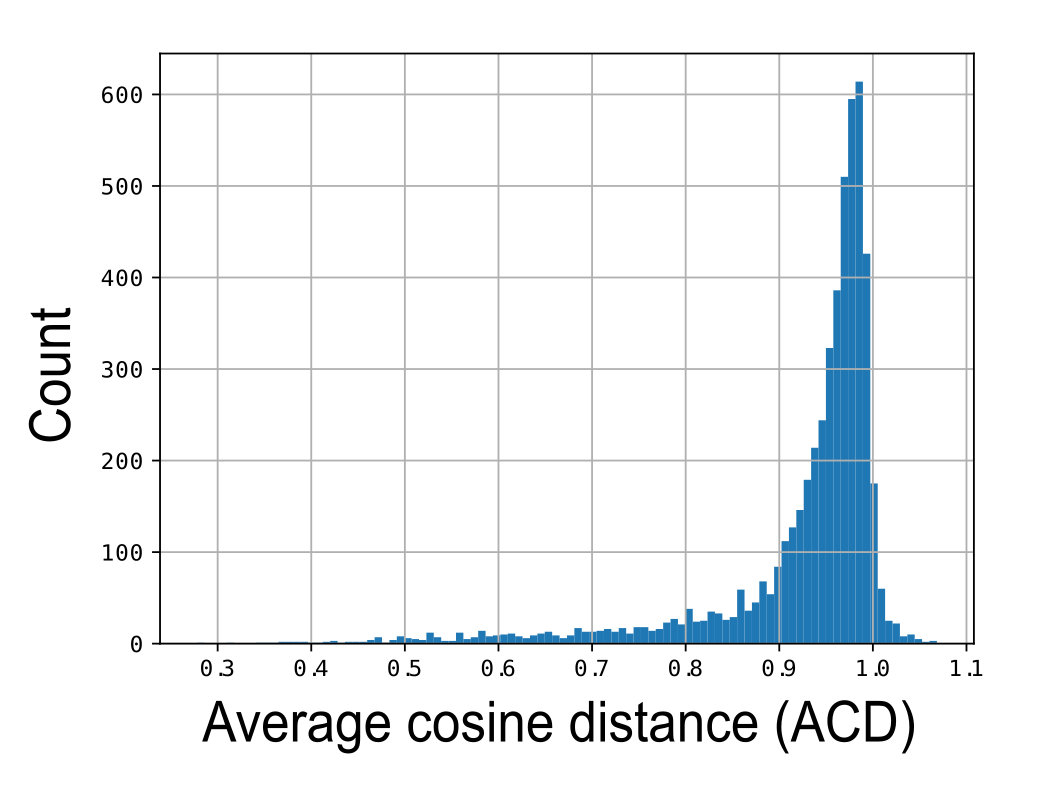


Figure S1. Distribution of average cosine distances of LINCS L1000 chemical signatures.


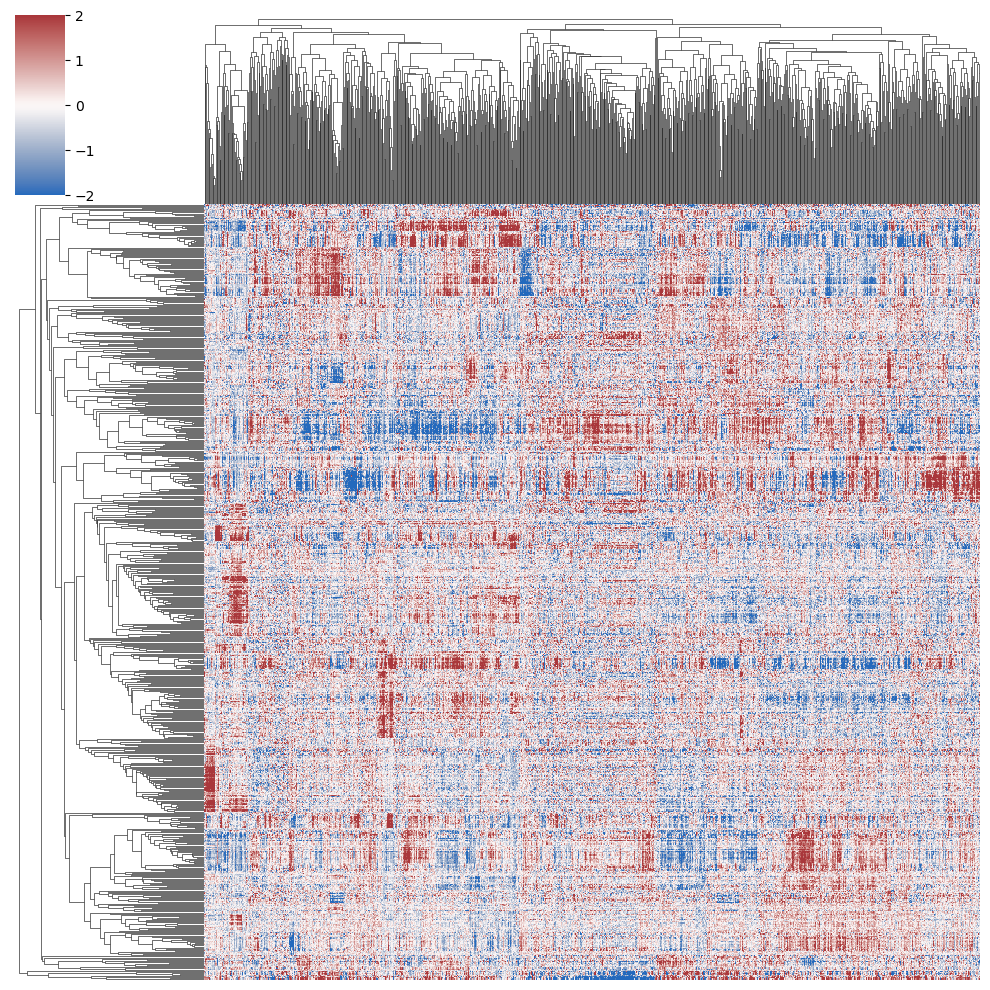


Figure S2. Cluster heatmap of the 297 compound signatures.

**Case Study 2**

We downloaded gene expression data of 38 primary cancer cell lines derived from different regions of gallbladder carcinoma tumors of seven patients from a previous study [1]. We normalized the data and created a cluster heatmap using the top 600 most varied genes. In performing the hierarchical clustering, we used z-score standardization along the column dimension, the average linkage method, and euclidean distance as the distance metric on both the row and column dimensions. After generating the JSON input to the DendroX app, we visualized the cluster heatmap in DendroX and highlighted two gene clusters and two cell line clusters. The results are shown in Figure S4. Based on the structure of the column dendrogram, the 38 cancer cell lines can be divided into two major clusters (left and right). Gene cluster 1 (C1 in blue) consists of genes that are mostly up-regulated in the left cell line cluster. Enrichment analysis on these genes show they are enriched in “Epithelial Mesenchymal Transition”, suggesting the cell lines in the left cluster are potentially more metastatic.


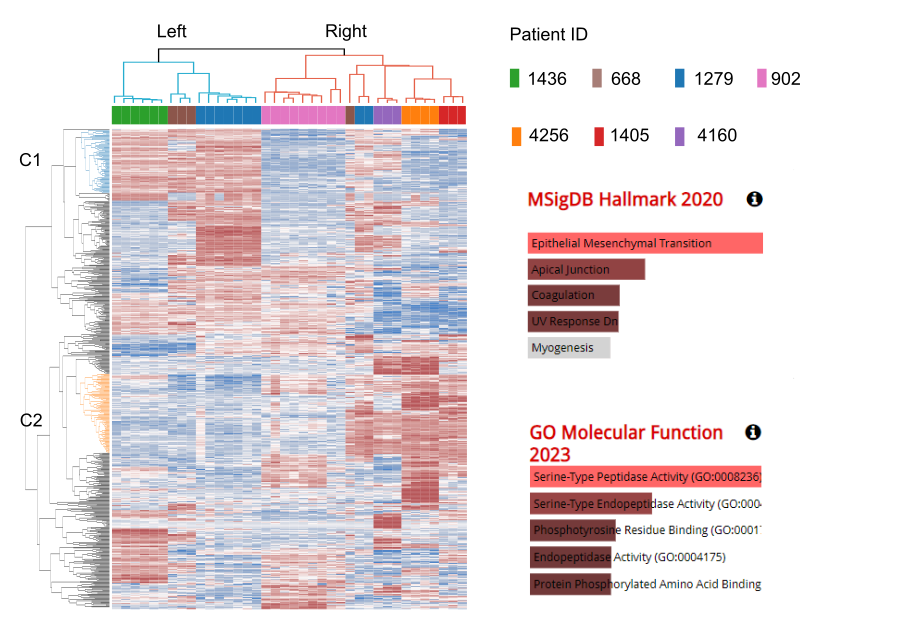


Figure S3. Cluster heatmap of the top 600 most varied genes of the 38 primary cancer cell lines. The color bar on the top indicates the patients from which they are derived.

An interesting observation in the cluster heatmap is that while the majority of cancer cell lines derived from patient 668 and 1279 are located in the left cluster, one cell line from 668 and two cell lines from 1279 are found in the right cluster next to each other. Gene cluster 2 (C2 in orange) comprises genes that are up-regulated in these three cell lines but not in those in the left cluster. Gene-set enrichment analysis showed that they are significantly enriched in “Serine-Type Peptidase Activity”. Serine protease activity has been implicated in tumor progression of multiple cancers of the epithelial origin [2, 3]. Users can review the case study by clicking the Genes example button in the input view in DendroX.

**Reference**

1. Feng F, Cheng Q, Li B, Liu C, Wang H, Li B, et al. Establishment and characterization of 38 novel patient-derived primary cancer cell lines using multi-region sampling revealing intra-tumor heterogeneity of gallbladder carcinoma. Human Cell. 2021;34:918–31.

2. Martin CE, List K. Cell surface–anchored serine proteases in cancer progression and metastasis. Cancer and Metastasis Reviews. 2019;38:357–87.

3. Steiro I, Vandsemb EN, Elsaadi S, Misund K, Sponaas A-M, Børset M, et al. The serine protease matriptase inhibits migration and proliferation in multiple myeloma cells. Oncotarget. 2022;13:1175–86.
